# Floral scent chemodiversity is associated with high floral visitor but low bacterial richness on flowers

**DOI:** 10.1111/nph.70600

**Published:** 2025-10-22

**Authors:** Maximilian Hanusch, Stefan Dötterl, Anne‐Amélie C. Larue‐Kontić, Alexander Keller, Robert R. Junker

**Affiliations:** ^1^ Department of Biology Marburg University 35042 Marburg Germany; ^2^ Department of Environment and Biodiversity Paris‐Lodron University Salzburg 5020 Salzburg Austria; ^3^ Faculty of Biology Ludwig Maximilians University Munich 82152 Planegg‐Martinsried Germany

**Keywords:** chemical diversity, floral microbiome, floral scent, multifunctional traits, plant–microbe interactions, pollination ecology

## Abstract

- Floral scents are complex blends of volatile compounds, yet the influence of floral scent chemodiversity, the richness, evenness, and functional disparity of phytochemical compounds in shaping interactions with flower visitors and microbes remains largely unexplored.
- Using a dataset of alpine plant species, we investigated how floral scent chemodiversity affects flower visitor and bacterial diversities on flowers.
- Our results reveal that high floral scent chemodiversity is associated with increased flower visitor richness but reduced bacterial richness on flowers. These findings led us to propose the ‘Filthy Pollinator Hypothesis’.
- Our hypothesis rests on two core ideas: flowers with chemodiverse scents attract a broader range of flower visitors, thereby increasing the potential for microbial transmission; and floral scent chemodiversity acts as a selective filter, mitigating the risks of unwanted microbial colonization by preventing the establishment of detrimental microbes while still allowing the establishment of a healthy microbiome. Floral scent chemodiversity may therefore not only shape the specialization/generalization of flower visitor assemblages but also act as a regulatory mechanism for microbial communities. By highlighting the multifunctionality of chemodiversity in structuring plant–animal and plant–microbe interactions, our study advances the understanding of chemodiversity and underscores its importance in plant ecology.

## Introduction

Since Fraenkel's seminal work (Fraenkel, 1959) on the reasons for the existence of phytochemicals, more than a dozen hypotheses have been proposed to explain the enormous *chemodiversity*, that is, the richness, evenness, and functional disparity of phytochemical compounds produced by a plant or plant organ (Hartmann, 2007; Wetzel & Whitehead, 2020; Petrén *et al*., 2024; Thon *et al*., 2024; Wittmann & Bräutigam, 2024). While the proposed hypotheses are not mutually exclusive and address different phytochemical patterns at various scales and levels of organization (Wetzel & Whitehead, 2020), most, if not all, of them share a common premise: higher levels of chemodiversity influence a wider range of recipients – whether they are herbivores, pollinators, or microbes. Despite the long‐standing interest in the causes of plant chemodiversity, only a few studies have tested the ecological function of chemodiversity as a trait on its own and not merely as a function of individual compounds (e.g., Whitehead *et al*., 2021; Sasidharan *et al*., 2023; Ziaja & Müller, 2023; Petrén *et al*., 2024).

Although its evolutionary origins remain debated (Moore *et al*., 2014; Wittmann & Bräutigam, 2024), chemodiversity is increasingly acknowledged as a crucial but underexplored component of a plant's functional phenotype (Wetzel & Whitehead, 2020; Müller & Junker, 2022; Petrén *et al*., 2023, 2024; Thon *et al*., 2024). While diversity itself is a multifaceted concept, chemodiversity has most commonly been quantified as a function of the richness and evenness of chemical compounds (Petrén *et al*., 2024). Methodological advancements now facilitate the incorporation also of the chemical disparity of compounds into the calculation of chemodiversity (Petrén *et al*., 2023). Disparity captures structural or biosynthetic differences among individual compounds, under the assumption that these differences potentially correspond to functional variation between compounds. This approach acknowledges the potential for additive and synergistic effects between compounds, especially those of high structural dissimilarity, which may give rise to novel ecological functions that extend beyond those of individual compounds (Liu & Zhao, 2016; Richards *et al*., 2016; Philbin *et al*., 2022). Although such insights have begun to illuminate the functional significance of chemodiversity, our broader ecological understanding of its consequences is still developing (Gershenzon & Dudareva, 2007; Petrén *et al*., 2024).

Plants are embedded in complex biotic communities in which they must selectively filter and structure interactions with other organisms (Junker, 2016). Beyond its role in enabling plants to respond to environmental changes such as temperature variation or increased UV exposure (Farré‐Armengol *et al*., 2014; Volf *et al*., 2022), the potential of chemodiversity in shaping interactions between plants and associated organisms has been shown to be of particular ecological importance (Whitehead *et al*., 2021; Wittmann & Bräutigam, 2024). In the context of pollination, for instance, individual floral scent compounds are well known to affect interactions with floral visitors by selective attractance or repellence (Raguso, 2008b; Junker & Blüthgen, 2010; Dötterl & Gershenzon, 2023). Moreover, studies have indicated that floral scent chemodiversity is closely linked to a plant's pollination strategy, whether attracting a diverse range of pollinators or targeting only a few, with higher chemodiversity typically attracting a broader range of pollinators (Burkle & Runyon, 2019; Benvenuti *et al*., 2020). These findings underscore the importance of floral scent chemodiversity in mediating plant–animal interactions and determining the degree of generalization/specialization in plant–pollinator relationships (Raguso, 2008a). However, whether the functional role of floral scent chemodiversity extends to structuring interactions with other trophic groups, such as flower‐inhabiting microorganisms, remains unclear.

Flower‐inhabiting microorganisms influence plant health, development, and reproduction in both positive and negative ways. On one hand, the floral microbiome is increasingly recognized as a coactive partner, contributing to critical functions such as processing and protecting floral resources for pollinators (Schaeffer *et al*., 2017), or modulating floral attractiveness, with implications for pollinator visitation (Herrera *et al*., 2009; Helletsgruber *et al*., 2017; Schaeffer *et al*., 2019; Keller *et al*., 2021; Steffan *et al*., 2024). On the other hand, microorganisms can also negatively impact plants by exhibiting pathogenic traits (Vannette, 2020), compromising floral resource integrity (Ngugi & Scherm, 2006), or disrupting beneficial interactions with pollinators (Good *et al*., 2014; Junker *et al*., 2014; Rering *et al*., 2018). Consequently, maintaining a healthy and functionally stable floral microbiome is essential for sustaining beneficial microbe‐mediated processes and maintaining plant fitness. The role of plant volatiles in shaping the plant microbiome has thus become an increasingly studied area of research (Junker & Tholl, 2013; Aleklett *et al*., 2014; Wei *et al*., 2022). While individual floral scent compounds have been shown to modulate microbial activity and composition (Junker *et al*., 2011; Burdon *et al*., 2018; Hammerbacher *et al*., 2019), it remains unknown whether overall chemodiversity exerts a structuring effect on microbial assemblages.

In this study, we examine the relationships between floral scent chemodiversity, flower visitor richness, and floral bacterial richness across 39 alpine plant species in a natural environment. We calculated the functional chemodiversity of floral scents using the R package chemodiv v.0.3.0 (Petrén *et al*., 2023) that quantifies functional chemodiversity by integrating both the quantitative composition and structural properties of floral scent compounds, enabling a more ecologically meaningful assessment of floral scent variation (Junker, 2018; Cosmo *et al*., 2021; Petrén *et al*., 2024). Additionally, we compared the relationships of chemodiversity with those of morphological floral traits for the same plant species to assess whether the richness of flower visitors and bacterial colonizers is more strongly associated with scent diversity or morphological variation. By integrating chemical and morphological perspectives, we provide new insights into the ecological role of floral scent chemodiversity in shaping both the specialization/generalization spectrum of flower visitor assemblages and the regulation of microbial communities on flowers. Our study connects the chemical diversity of floral scent to two simultaneous challenges: attracting pollinators and controlling microbial colonization. This perspective highlights the multifunctionality of floral scent and its central role in structuring both plant–animal and plant–microbe interactions.

## Materials and Methods

### Research area and sampling procedure

We conducted fieldwork in seven alpine plant communities at the Grossglockner Mountain, Austrian Alps, ranging from 1146 to 2750 m above sea level during May, June, and July 2014. Communities were selected based on accessibility and their dominant coverage by pastures. We assessed the relationships between floral scent chemodiversity, flower visitor richness, and floral bacterial richness across *n* = 92 individuals representing 39 alpine plant species, and measured morphological floral traits of all these species. Due to destructive sampling, flower visitors, floral scent, microbial communities, and floral traits were measured on different subsets of individuals. All observations, however, were collected within the same plant communities under comparable environmental conditions.

### Floral scent sampling

Floral volatiles were collected using dynamic headspace sampling methods directly in the field (Larue *et al*., 2016; Larue‐Kontić & Junker, 2016). Single inflorescences were enclosed in polyethylene tetraphthalate (PET) oven bags (Toppits® Cofresco Fischhalteprodukte GmbH & Co. KG, Minden, Germany) for 45 min, and afterward, the enriched headspace was sampled for 2 min into volatile traps with a flow rate of 200 ml min^−1^ by the use of a rotary vane pump (G12/01 EB; Rietschle Thomas Inc., Puchheim, Germany). The scent traps were filled with a mixture of 1.5 mg Tenax‐TA (mesh 60–80; Supelco, Germany) and 1.5 mg Carbotrap B (mesh 20–40; Supelco, Germany). Floral scent bouquets were analyzed using an automatic thermal desorption system (TD, model TD‐20, Shimadzu, Japan) coupled with gas chromatography/mass spectroscopy (GC‐MS, model QP2010 Ultra EI, Shimadzu, Japan). The gas chromatograph was equipped with a ZB‐5 column (Zebron ZB‐5, 5% phenyl polysiloxane, length 60 m, inner diameter 0.25 mm, film thickness 0.25 μm, Phenomenex, Newport Beach, USA). Carrier gas flow (helium) was set to 1.5 ml min^−1^ and the oven temperature was held for 1 min at 40°C and then rose with 6°C min^−1^ until the maximum of 250°C was reached. The interface of the mass spectroscopy was 260°C and the ion trap worked at 200°C. Chromatograms and mass spectra were analyzed with the GCMSsolutions software (v.2.72, Shimadzu Corp.). All peaks, which occurred in the scent samples but not in ambient air control samples, were classified as floral volatiles. Compounds were tentatively identified by comparison of linear retention indices (RI, based on a series of commercially available n‐alkanes C7‐C20; Van Den Dool & Kratz, 1963) and a match of mass spectra to spectra available in the databases ADAMS, ESSENTIALOILS‐23P, FFNSC 2, and W9N11. If possible, compound identities were verified using RI and mass spectra of authentic standards available in the Plant Ecology Lab of the Paris‐Lodron University of Salzburg. The relative amount of compounds (contribution of single compounds to total peak area of a sample) was determined and used for statistical analyses (refer to the following section). All identified compounds were compared against the PubChem database to retrieve corresponding SMILES strings and InChIKeys for specific chemical structures. The abundance of floral volatiles per sample and the list of all identified compounds are provided in Supporting Information Datasets S1 and S2, respectively.

### Morphological traits

The selection of floral traits comprised morphological features known to influence plant–pollinator interactions (Junker & Parachnowitsch, 2015; Michelot‐Antalik *et al*., 2025). For each plant species and plot, we measured five floral traits on *n* = 7 individuals per species: display size of flowers, nectar tube width, nectar tube depth, position of pollen relative to the flower surface (negative values indicating anthers located inside the nectar tube), and flower inclination (vertical orientation). Flower display size, nectar tube width and depth, and pollen position were measured to the nearest 1 mm using a caliper. Flower inclination was recorded in the field using a triangle ruler. Samples were collected in the field, stored in a cooler, and transported to the laboratory, where measurements began within 1 h of collection. For finer resolution, morphological measurements were conducted under a binocular microscope (Leica Microsystems, Vienna, Austria). An overview of mean values for measured traits is provided in Dataset S3.

### Flower visitor sampling

Flower visitor interactions were recorded on sunny days between 8:45 and 18:00. Observations were conducted by walking slowly along transects, collecting all insects observed contacting reproductive parts of flowers. Each transect was observed three times per month throughout the field season, with sessions lasting 24 min, resulting in 720 min of observation time per transect. Sampling followed a randomized order to minimize temporal biases, and up to two observers worked simultaneously during fieldwork. All collected insects were stored in a freezer for later identification. Due to limitations in species‐level identification, flower visitor richness was calculated at the morphospecies level, with a comprehensive list of observed insects provided in Dataset S4. In total, *n* = 656 flower visitor interactions were recorded, and the relationship between chemodiversity and flower visitor richness was assessed in a subset of *n* = 53 individuals representing 31 plant species.

### Microbial sampling

We sampled bacteria inhabiting floral tissues during peak flowering of the vegetation (20 May to 05 July 2014). For every plant sample, we took 1–5 flowers or floral units according to different flower sizes of the species to ensure that the size of the samples was largely consistent among species. Immediately after collection, each sample was placed into ZR BashingBeads Lysis tubes pre‐filled with 750 μl of DNA/RNA Shield lysis solution (ZymoBIOMICS DNA Miniprep Kit; Zymo Research, Irvine, CA, USA). To minimize contamination, floral material was collected using sterilized forceps (treated with 70% ethanol and flamed). Before DNA extraction, samples were sonicated and plant tissue removed to reduce the proportion of plant‐derived DNA. The samples were then processed in a ball mill at 30.0 s^−1^ for 9 min. Microbial DNA was isolated using the ZymoBIOMICS DNA Miniprep Kit according to the manufacturer's protocol. We amplified the V4 region of the 16S rRNA gene using a single‐PCR and dual‐indexing approach following the protocol by (Kozich *et al*., 2013). This method employs barcoded primers incorporating an Illumina adapter, index sequence, pad, and linker, followed by the gene‐specific primers 515f (5′‐GTGCCAGCMGCCGCGGTAA‐3′) and 806r (5′‐GGACTACHVGGGTWTCTAAT‐3′). PCR amplification was performed in triplicate (3 × 10 μl) using Phusion Plus PCR Master Mix (Thermo Scientific, Waltham, MA, USA), following the thermal cycling conditions: initial denaturation at 98°C for 30 s; 30 cycles of 98°C for 10 s, 55°C for 10 s, and 72°C for 30 s; with a final extension at 72°C for 5 min. Reactions were prepared according to the pipetting scheme described by Sickel *et al*. (2015).

Amplification success was verified using a 96‐well 1% agarose SYBR Safe E‐Gel on an E‐Gel Power Snap Plus Electrophoresis Device (ThermoFisher Scientific). PCR products were normalized using SequalPrep Normalization Plates (Invitrogen) and pooled into four plate‐specific libraries. Library quality and fragment size were assessed with a High Sensitivity DNA Chip on an Agilent 2100 Bioanalyzer. DNA concentrations were quantified using the 1× dsDNA HS Assay Kit on a Qubit 4 Fluorometer (ThermoFisher Scientific). The four plate pools were then combined equimolarly to a final concentration of 2 nM and sequenced on an Illumina MiSeq platform using paired‐end 2 × 250 bp chemistry with a 5% PhiX control spike‐in.

Raw sequencing data were processed using the metabarcoding pipeline available at https://github.com/chiras/metabarcoding_pipeline (Leonhardt *et al*., 2022). Forward and reverse reads were merged using Vsearch v.2.14.2 (Rognes *et al*., 2016), followed by quality filtering with an expected error threshold (EE < 1) as described by Edgar & Flyvbjerg (2015). Reads shorter than 170 bp and singletons were removed before downstream analysis. Amplicon sequence variants (ASVs) were inferred using VSEARCH, and *de novo* chimera removal was performed with UCHIME3 (Edgar, 2016b). Final ASVs were assigned taxonomy via global alignment (97% identity threshold) against the RDP (v.18), Greengenes (v.13.5), and SILVA (v.123) reference databases. Remaining unclassified reads were further annotated using Sintax (Edgar, 2016a) with a confidence threshold of 0.9 against the RDP (v.18) database. Initial data preprocessing was conducted in R using the phyloseq v.1.48.0 package. Reads assigned to unresolved taxa or non‐bacterial groups (e.g. algae, fungi, and mitochondria) were removed, and samples were subset to include only bacterial taxa. Processed microbial community data are provided in Dataset S5, and the relationship between chemodiversity and bacterial richness was assessed in a subset of *n* = 39 individuals representing 31 plant species.

### Calculation of functional chemodiversity

Chemodiversity was calculated using the chemodiv R package v.0.3.0 (Petrén *et al*., 2023), which is specifically designed to quantify the chemical diversity of complex mixtures such as floral scents. A total of *n* = 264 compounds were measured from the floral scent samples. Of these, *n* = 158 (60%) were successfully identified and annotated with SMILES strings and InChIKeys, which provide standardized representations of chemical structures and enable computational analysis of their properties. The remaining compounds, for which structural information was unavailable, were assigned average dissimilarity values to ensure their inclusion in the analysis without biasing the results.

We calculated functional scent diversity by calculating structural similarities among compounds using the flexible maximum common substructure (fmcs) algorithm (Wang *et al*., 2013). This method calculates pairwise structural dissimilarities among compounds by identifying the largest common substructures, reflecting biosynthetic or functional differences. For compounds that remained unidentified, we assigned the mean dissimilarity value to account for their unknown structures in the analysis. We used the resulting dissimilarity matrix to calculate functional Hill diversity, a metric that quantifies the effective total dissimilarity between compounds within a sample (Chiu & Chao, 2014), with the sensitivity parameter set to *q* = 1, a commonly used value that accounts for the relative contributions of individual compounds without overemphasizing rare or dominant ones. An overview of different aspects of chemodiversity, that is, chemical compound richness, compound evenness, and mean pairwise structural distances of chemical compounds per plant species, is provided as boxplots in Fig. S1.

### Statistical analysis

We assessed the effects of functional floral scent diversity on pollinator and bacterial richness using the glmer function in the R package lme4 (v.1.1‐35.5). For both response variables (flower visitor richness and bacterial ASV richness), we constructed and compared a set of five candidate models that differed in whether elevation was included as a fixed or as a random effect, and in whether the random effect of plant species identity was included (see Tables S1, S2 for full model comparison). Plant species, when included, were treated as a random factor to account for baseline interspecific variation in floral traits. Elevation was considered in both fixed and random forms to account for its known influence on ecological interactions in alpine environments (Minachilis *et al*., 2023; Ramakrishnan *et al*., 2024), while allowing flexibility in whether it explained consistent directional trends (fixed) or structured variation among sites at different elevations (random).

The best‐fitting models, selected based on model diagnostics and fit, included elevation as a fixed effect in both cases. Floral visitor richness showed no evidence of overdispersion (dispersion ratio = 0.88) and was therefore modeled using a Poisson GLMM. By contrast, bacterial ASV richness exhibited strong overdispersion under a Poisson model (dispersion ratio = 12.63), indicating greater variability in the data than expected under a Poisson distribution. To account for this, we used a negative binomial GLMM, which substantially improved model fit (dispersion ratio = 0.76). For ASV richness, the random effect of plant species identity was excluded from the final model due to boundary singularity, indicating negligible variance at the species level.

Both floral morphology and floral scent can influence how generalized or specialized a plant's interactions are (Johnson & Steiner, 2000; Fenster *et al*., 2004). Morphological traits determine whether floral resources are accessible to a wide range of flower visitors, and the microbiomes also depend on morphological characteristics (Aleklett *et al*., 2014). To evaluate whether morphology rather than floral scent drives specialization/generalization of plant species in our system, we compared the strength of associations between flower visitor richness, bacterial richness, floral scent chemodiversity, and morphological traits. For this, we used species‐mean values of morphological traits and chemodiversity, and calculated pairwise Spearman's rank correlation coefficients between variables. The resulting correlation matrix was visualized as a network, and we applied Louvain clustering (R Package igraph v.2.1.4) to identify modules, that is, groups of traits and community metrics that are more strongly associated with each other than with the rest of the network (Blondel *et al*., 2008; E‐Vojtkó *et al*., 2022).

## Results

**Fig. 1:**
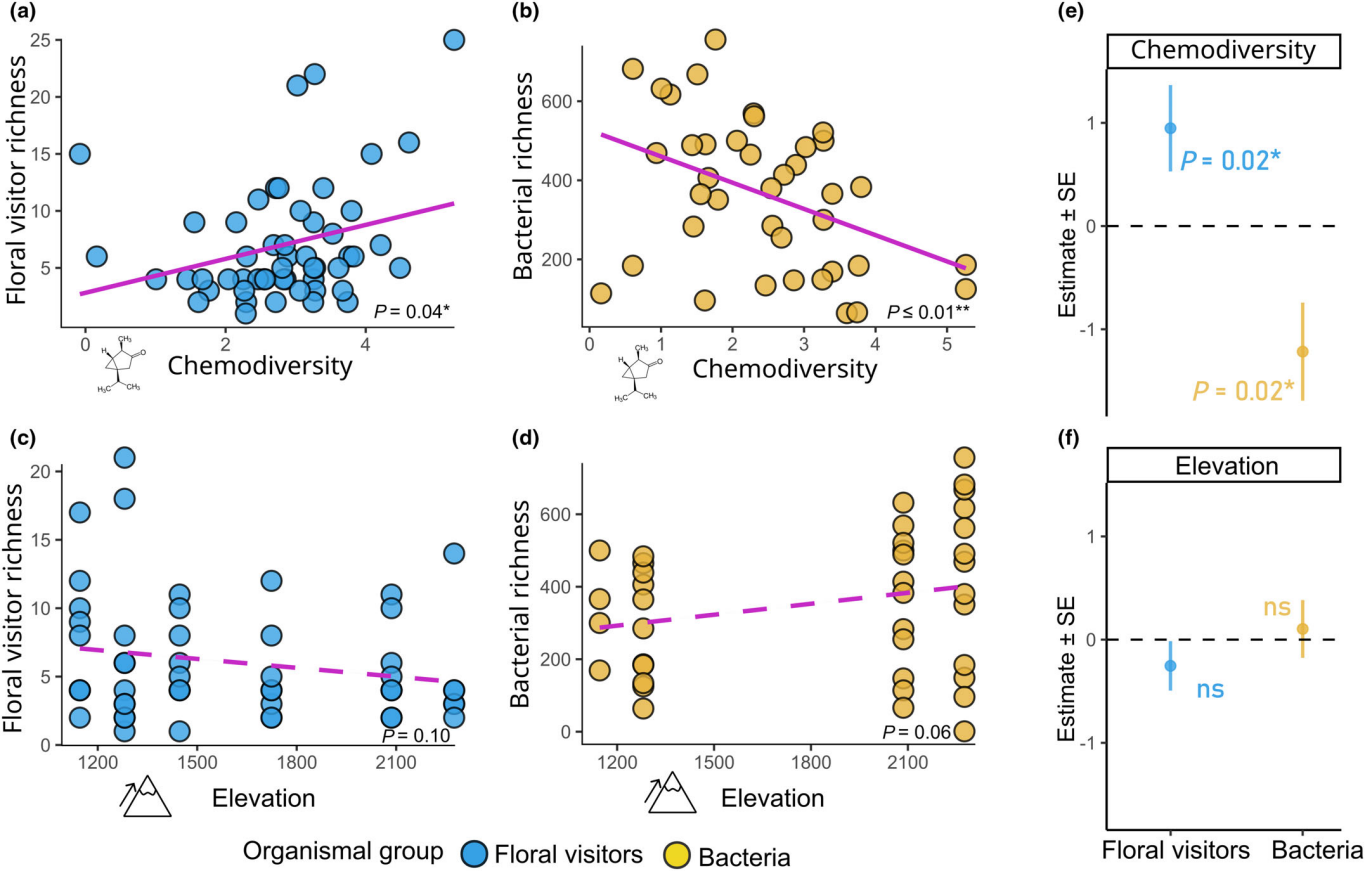
Relationships between (a) flower visitor richness and chemodiversity, showing a significant positive relationship; (b) bacterial richness and chemodiversity, showing a significant negative relationship; (c) flower visitor richness and elevation, showing no significant relationship; and (d) bacterial richness and elevation, showing no significant relationship. Each point represents a raw observation from an individual plant‐flower sample, with some plant species represented multiple times. Linear trends (lines) and significance annotations are based on simple linear models fitted to all data points and are shown for visualization purposes only. No correction for species identity or repeated measures was applied. Panels (e, f) summarize estimated effect sizes (mean ± SE) from generalized linear mixed effect models for (e) chemodiversity effects on richness (corrected for elevation and plant species) and (f) elevation effects on richness (corrected for chemodiversity and plant species). Note that positive and negative effect directions correspond to the directionality of the slope estimates in (a–d).

Floral scent chemodiversity had taxon‐specific effects on the diversity of flower‐associated communities (Fig. 1a,b,e). Flowers emitting more chemically diverse scents attracted a greater richness of flower visitors (GLMM: incidence rate ratio, IRR = 2.49; 95% CI = 1.17–5.29; *P* = 0.02), aligning with previous findings (Burkle & Runyon, 2019; Benvenuti *et al*., 2020). By contrast, increased chemodiversity was associated with lower bacterial richness on flowers (IRR = 0.37; 95% CI = 0.18–0.90; *P* = 0.02), indicating that floral scent chemodiversity acts as a selective filter on microbial communities. Elevation had no significant effect on either pollinator (IRR = 0.72; 95% CI = 0.46–1.12; *P* = 0.15) or bacterial richness (IRR = 1.24; 95% CI = 0.79–1.91; *P* = 0.34), indicating that the observed patterns are primarily driven by floral scent chemodiversity rather than environmental factors (Fig. 1c,d,f).

**Fig. 2:**
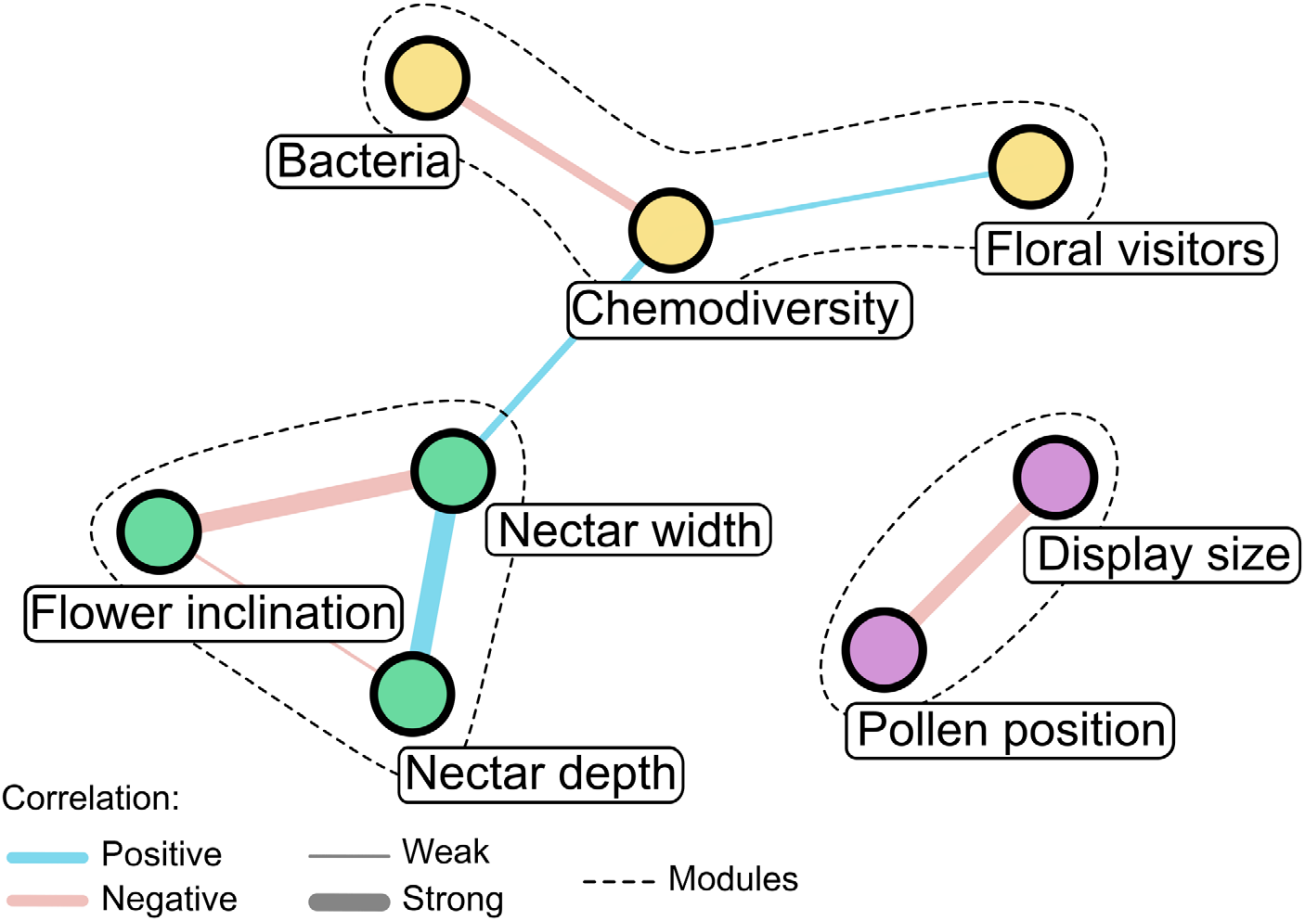
Network of significant pairwise associations among morphological floral traits, floral scent chemodiversity, bacterial richness, and flower visitor richness, based on Spearman's rank correlation coefficients. Edge color indicates the direction of correlation (blue = positive, red = negative), and edge thickness represents correlation strength. Node color indicates module membership as identified by Louvain clustering; dashed lines outline the detected modules.

Chemodiversity showed a positive monotonic relationship with nectar tube width (rho = 0.39, *P* = 0.01) and floral visitor richness (rho = 0.36, *P* = 0.03), and a negative monotonic relationship with bacterial richness (rho = −0.44, *P* < 0.01). Network analysis revealed that morphological traits were grouped into two separate modules. By contrast, floral scent chemodiversity, bacterial richness, and flower visitor richness formed a distinct module, indicating stronger associations among these variables than with any other trait (Fig. 2; Table S3).

## Discussion

Our findings reveal that floral scent chemodiversity, rather than morphological flower traits, is linked to both floral visitor richness and bacterial richness. Previous work has emphasized that specific micro‐niche characteristics, such as the structural configuration of floral organs, can strongly influence the diversity and composition of floral microbiomes (Aleklett *et al*., 2014; Taneda *et al*., 2015; Gaube *et al*., 2021). Our results suggest that floral scent chemodiversity functions as an additional regulator of flower‐associated microbial communities (Junker & Tholl, 2013; McArt *et al*., 2014; Vannette, 2020; Gaube *et al*., 2023). Similarly, floral morphology is widely recognized as a key determinant of flower visitor communities, with traits such as organ placement or overall display size defining which pollinators can effectively exploit a flower (Fenster *et al*., 2004; Michelot‐Antalik *et al*., 2025). However, in our dataset, morphological traits did not show significant associations with either flower visitor or bacterial richness, and thus the degree of specialization/generalization of alpine plant species. We note that our study did not include flower color, which, although important in some systems, is often less informative than morphological traits in pollination ecology (Dellinger, 2020). Nevertheless, color can interact with scent in shaping pollinator assemblages, and such multimodal effects warrant further investigation (Junker & Parachnowitsch, 2015; Kantsa *et al*., 2017, 2018; Shen *et al*., 2024).

Floral scent chemodiversity showed contrasting relationships with flower visitor and bacterial richness: higher chemodiversity is linked to more diverse flower visitor assemblages, yet lower bacterial diversity on flowers. One possible explanation for this contrast may be the close links between flower visitors and the microbes they transmit among flowers. Flower visitors are well known to serve as microbial vectors between flowers, with different species carrying a distinct set of microorganisms (de Vega *et al*., 2021), thereby actively shaping floral microbial assemblages (Durrer & Schmid‐Hempel, 1994; McFrederick *et al*., 2017; Vannette *et al*., 2021; Luizzi *et al*., 2024). In this context, it is assumed that flowers function as hubs of microbial transmission where microbes can accumulate and be further dispersed (Keller *et al*., 2021; Argueta‐Guzmán *et al*., 2025). If greater floral scent chemodiversity attracts a broader range of pollinators, higher microbial transfer rates via pollinator visitation should lead to a higher number of bacterial taxa on flowers of generalist plant species (Vannette & Fukami, 2017). However, we observed an opposite pattern: chemically diverse and therefore more generalized flowers (in terms of flower visitation) harbored lower bacterial richness.

Building on these insights, we propose the ‘Filthy Pollinator Hypothesis’, highlighting the role of flower visitors not only as mutualistic agents of pollen transfer, but also as carriers of a diverse and ecologically consequential microbial load. Our hypothesis rests on two core ideas: first, flowers with chemically diverse scents tend to be pollination generalists, attracting a broad range of pollinator species (in line with the Interaction Hypothesis of chemodiversity; Wetzel & Whitehead, 2020). Second, beyond maximizing pollinator attraction, floral scent chemodiversity functions as a selective filter helping to maintain a stable and healthy floral microbiome, which in turn can support plant fitness and ecosystem functioning. Although animal vectors are not the sole source of microbial influx on flowers (Vokou *et al*., 2012; Sessitsch *et al*., 2023), they are directed vectors of microbes between flowers, many of which can be pathogenic (McArt *et al*., 2014). As different pollinator species carry distinct microbial assemblages (Ushio *et al*., 2015; Ambika Manirajan *et al*., 2016), generalist plants are likely to experience a more diverse and unpredictable microbial influx on their floral surfaces. However, rather than passively acquiring a random assemblage of microbes introduced by pollinators, flowers actively shape their microbial communities through selective filtering by favoring microbes tolerant to the diverse chemical environment while limiting colonization by others. This filtering results in plant species‐ and organ‐specific microbial communities that are not primarily shaped by passive transmission and colonization but rather by strong selective mechanisms (Massoni *et al*., 2020; Gaube *et al*., 2021; Qian *et al*., 2021; Ramakrishnan *et al*., 2024; Cecala *et al*., 2025).

Floral scents are known to possess individual chemical compounds that have constitutive antimicrobial properties (Thornburg *et al*., 2003; Gershenzon & Dudareva, 2007; Sasu *et al*., 2010; Huang *et al*., 2012; Hammerbacher *et al*., 2019). For example, some compounds disrupt bacterial cell walls (Eshboev *et al*., 2024), while others interfere with DNA replication and protein synthesis (Šovljanski *et al*., 2023), or energy production (Mickymaray, 2019). More diverse floral scents may function as broad‐spectrum antimicrobial agents, regulating microbial communities through the combined effects of multiple volatile compounds. This aligns with the interaction or synergy hypothesis of chemodiversity, which posits that chemically diverse mixtures enhance ecological functions beyond what individual compounds achieve alone (Richards *et al*., 2016; Wetzel & Whitehead, 2020; Putra & Müller, 2023). Moreover, the broad‐spectrum antimicrobial properties of floral chemodiversity may serve as a generalized defense strategy, helping plants keep pace with evolving phytopathogens in a dynamic coevolutionary arms race (Firn & Jones, 2003). This mechanism could also provide an evolutionary explanation for the persistence of floral scent chemodiversity, as predicted by modeling approaches (Wittmann & Bräutigam, 2024).

Our study advances the ecological understanding of floral scent chemodiversity by highlighting its role in mediating both plant–animal and plant–microbe interactions. Within the framework of the ‘Filthy Pollinator Hypothesis’, we propose that floral chemodiversity not only modulates generalization/specialization in plant–animal interactions by influencing pollinator attraction across species but also acts as a selective filter influencing the composition of flower‐associated microbial communities. Pollinators introduce a diverse array of microbes to flowers, including pathogenic and non‐pathogenic symbionts as well as transient taxa (McFrederick *et al*., 2012; Graystock *et al*., 2015; Figueroa *et al*., 2019), but only a subset is likely to persist in the chemically selective conditions of the floral niche (Müller & Junker, 2022). Thus, chemodiversity influences which microbes persist by creating a chemically complex environment, limiting the establishment of less compatible taxa. Importantly, compatibility with the floral niche does not imply mutualism, as both benign and pathogenic microbes can be chemically well adapted to chemodiverse floral environments. Nonetheless, floral scent chemodiversity contributes to the stabilization of the floral microbiome by selectively shaping microbial communities, with important implications for plant health and reproductive success.

Furthermore, our findings suggest that generalization in pollinator attraction may come with a trade‐off: a greater need for microbial filtering to sustain a stable floral microbiome, suggesting that not only flower‐visiting animals but also microorganisms select for floral traits. To test this hypothesis, we suggest to experimentally investigating the direct effects of floral scent chemodiversity on pollinator attraction and microbial colonization under controlled conditions. In particular, experimental manipulations of the three main components of diversity, richness, evenness, and disparity could provide deeper insights into the mechanisms driving these interactions. Expanding such research across diverse plant species and environmental contexts will not only refine our understanding of plant–microbe‐pollinator interactions but also offer broader evolutionary and ecological insights into the functional role of chemodiversity in shaping plant interactions and biodiversity.

## Competing interests

None declared.

## Author contributions

RRJ initiated the study. A‐ACL‐K and RRJ sampled the data. RRJ, A‐ACL‐K, AK, SD and MH processed the data. MH and RRJ performed the analysis using inputs from A‐ACL‐K, AK and SD. MH and RRJ wrote the first draft of the manuscript. All authors contributed to manuscript revision and approved the final version.

## Disclaimer

The New Phytologist Foundation remains neutral with regard to jurisdictional claims in maps and in any institutional affiliations.

## Supporting information


**Dataset S1** Quantitative floral scent samples.

**Dataset S2** Compounds identified in floral scent samples.

**Dataset S3** Morphological flower traits.

**Dataset S4** Quantitative floral visitor sampling.

**Dataset S5** Bacterial communities detected on flowers.

**Fig. S1** Distribution of per‐species mean values for six metrics of chemodiversity and biotic interactions.

**Table S1** Model comparison for ASV richness.
**Table S2** Model comparison for floral visitor richness.
**Table S3** Pairwise Spearman correlations and *P*‐values among chemodiversity, flower visitor richness, bacterial richness, and morphological flower traits.
Please note: Wiley is not responsible for the content or functionality of any Supporting Information supplied by the authors. Any queries (other than missing material) should be directed to the *New Phytologist* Central Office.

## Data Availability

The data that supports the findings of this study are available in Datasets [Link], [Link], [Link], [Link], [Link] of this article.
