# Supplementary material for: Floral scent chemodiversity is associated with high floral visitor but low bacterial richness on flowers
Source: New Phytol. 2025 Oct 22;248(6):3270–9. doi: 10.1111/nph.70600 (PMC12630430; doi:10.1111/nph.70600)

New Phytologist Supporting Information

Article title: Floral scent chemodiversity is associated with high floral visitor but low bacterial richness on flowers

Authors: Maximilian Hanusch[1], Stefan Dötterl[2], Anne-Amélie C. Larue-Kontić[2], Alexander Keller[3], Robert R. Junker[1]

[1]Philipps-University Marburg, Evolutionary Ecology of Plants, Germany, 35042 Marburg
[2]Paris-Lodron University Salzburg, Department of Environment and Biodiversity, Austria, 5020 Salzburg
[3]Ludwig Maximilians University Munich, Faculty of Biology, Germany, 82152 Planegg-Martinsried

Article acceptance date: 11 September

Fig. S1. Boxplots showing the distribution of per-species mean values for six metrics: Chemodiversity, Chemical richness, Chemical evenness, Mean pairwise distance, ASV richness, and Floral visitor richness. Each point represents the mean value for one plant species, calculated across all available samples.

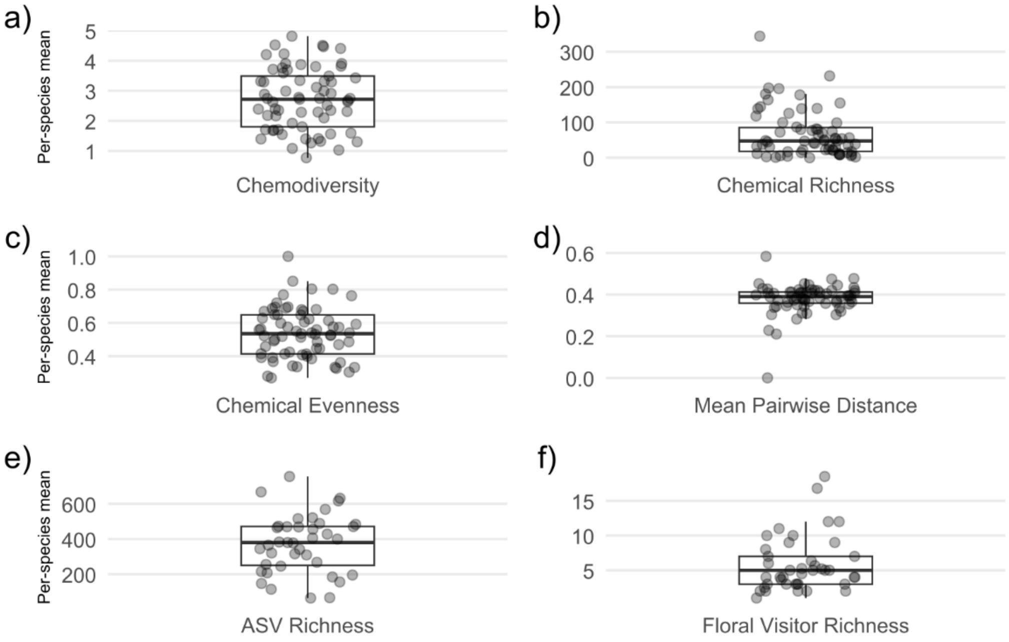

Supplement: Supplementary file 6 — Fig. S1 Distribution of per‐species mean values for six metrics of chemodiversity and biotic interactions. [file NPH-248-3270-s002.pdf]
