# Supplementary material for: Floral scent chemodiversity is associated with high floral visitor but low bacterial richness on flowers
Source: New Phytol. 2025 Oct 22;248(6):3270–9. doi: 10.1111/nph.70600 (PMC12630430; doi:10.1111/nph.70600)
Supplement: Supplementary file 7 — Table S1 Model comparison for ASV richness. Table S2 Model comparison for floral visitor richness. Table S3 Pairwise Spearman correlations and P‐values among chemodiversity, flower visitor richness, bacterial richness, and morphological flower traits. Please note: Wiley is not responsible for the content or functionality of any Supporting Information supplied by the authors. Any queries (other than missing material) should be directed to the New Phytologist Central Office. [file NPH-248-3270-s001.pdf]

New Phytologist Supporting Information

Article title: Floral scent chemodiversity is associated with high floral visitor but low bacterial richness on flowers

Authors: Maximilian Hanusch[1], Stefan Dötterl[2], Anne-Amélie C. Larue-Kontić[2], Alexander Keller[3], Robert R. Junker[1]

[1]Philipps-University Marburg, Evolutionary Ecology of Plants, Germany, 35042 Marburg
[2]Paris-Lodron University Salzburg, Department of Environment and Biodiversity, Austria, 5020 Salzburg
[3]Ludwig Maximilians University Munich, Faculty of Biology, Germany, 82152 Planegg-Martinsried

Article acceptance date: 11 September

Table S1. Model comparison for ASV richness. Five competing models were fitted using combinations of floral chemodiversity, elevation, and plant species as fixed or random effects. Models were evaluated using the {performance} R package. Goodness of fit measures: RMSE (lower is better), σ or residual standard deviation (lower is better), Score log and Score spherical (higher is better). Variance explained was assessed with R^2 conditional, R^2 marginal, and R^2 Nagelkerke (all higher is better), and the intraclass correlation coefficient ICC (higher values indicate more variance explained by grouping factors). Information theoretic weights AIC wt, AICc wt, and BIC wt indicate the relative support for each model, where values closer to 1 are better. The Performance Score is a composite ranking from, scaled between 0 for the worst and 1 for the best, integrating all criteria.

ASV Models												
**Description**	**Model Fit**				**R2 conditional**	**R2 marginal**	**ICC**	**R2 Nagelkerke**	**Weights**			**Performance Score**
	**RMSE**	**Sigma**	**Score log**	**Score spherical**					**AIC wt**	**AICc wt**	**BIC wt**	
NegBinom GLM: Chemodiversity + Elevation (no random effects)	170.107	1	-6.62	0.144	NA	NA	NA	0.263	0.624	0.696	0.792	0.90%
NegBinom GLMM: Chemodiversity + Elevation + (1|Plant)	170.107	3.54	- 6.611	0.144	NA	NA	NA	NA	0.23	0.186	0.127	0.40%
NegBinom GLMM: Chemodiversity + (1|Elevation) + (1|Plant)	172.798	3.465	- 6.612	0.144	NA	NA	NA	NA	0.146	0.119	0.081	0.40%
Poisson GLMM: Chemodiversity + (1|Elevation) + (1|Plant)	65.888	1	- 9.656	0.129	0.995	0.262	0.993	NA	0	0	0	0.30%
Poisson GLMM: Chemodiversity + Elevation + (1|Plant)	66.337	1	- 9.739	0.127	0.995	0.273	0.993	NA	0	0	0	0.30%

Table S2. Model comparison for floral visitor richness. Five competing models were fitted using combinations of floral chemodiversity, elevation, and plant species as fixed or random effects. Models were evaluated using the {performance} R package. Goodness of fit measures: RMSE (lower is better), σ or residual standard deviation (lower is better), Score log and Score spherical (higher is better). Variance explained was assessed with R^2 conditional, R^2 marginal, and R^2 Nagelkerke (all higher is better), and the intraclass correlation coefficient ICC (higher values indicate more variance explained by grouping factors). Information theoretic weights AIC wt, AICc wt, and BIC wt indicate the relative support for each model, where values closer to 1 are better. The Performance Score is a composite ranking from, scaled between 0 for the worst and 1 for the best, integrating all criteria.

Floral Visitor Models												
**Description**	**Model Fit**				**R2 conditional**	**R2 marginal**	**ICC**	**R2 Nagelkerke**	**Weights**			**Performance Score**
	**RMSE**	**Sigma**	**Score log**	**Score spherical**					**AIC wt**	**AICc wt**	**BIC wt**	
Poisson GLMM: Chemodiversity + Elevation + (1|Plant)	2.408	1	-2.173	0.128	0.615	0.12	0.563	NA	0.53	0.56	0.641	1.00%
Poisson GLMM: Chemodiversity + (1|Elevation) + (1|Plant)	2.461	1	-2.193	0.127	NA	NA	NA	NA	0.186	0.197	0.225	0.70%
NegBinom GLMM: Chemodiversity + Elevation + (1|Plant)	2.413	767.042	-2.564	0.125	0.613	0.12	0.56	NA	0.203	0.172	0.092	0.30%
NegBinom GLMM: Chemodiversity + (1|Elevation) + (1|Plant)	2.467	663.528	-2.557	0.126	NA	NA	NA	NA	0.073	0.062	0.033	0.30%
NegBinom GLM: Chemodiversity + Elevation (no random effects)	4.424	1	-2.731	0.126	NA	NA	NA	0.306	0.008	0.008	0.009	0.20%

Table S3. Pairwise Spearman correlations and p-values among Chemodiversity, flower visitor richness, bacterial richness, and morphological flower traits.

Variable 1	Variable 2	rho	p
Flower visitor richness	Chemodiversity	0.36	0.032
Bacterial richness	Chemodiversity	-0.44	0.008
Flower inclination	Chemodiversity	-0.20	0.216
Nectar depth	Chemodiversity	0.13	0.417
Nectar width	Chemodiversity	0.39	0.011
Pollen position	Chemodiversity	0.16	0.326
Display size	Chemodiversity	0.04	0.780
Bacterial richness	Flower visitor richness	-0.01	0.966
Flower inclination	Flower visitor richness	-0.17	0.428
Nectar depth	Flower visitor richness	0.13	0.542
Nectar width	Flower visitor richness	0.13	0.547
Pollen position	Flower visitor richness	-0.28	0.179
Display size	Flower visitor richness	0.28	0.176
Flower inclination	Bacterial richness	-0.27	0.187
Nectar depth	Bacterial richness	0.14	0.498
Nectar width	Bacterial richness	-0.04	0.852
Pollen position	Bacterial richness	0.11	0.590
Display size	Bacterial richness	-0.11	0.588
Nectar depth	Flower inclination	-0.32	0.024
Nectar width	Flower inclination	-0.59	0.000
Pollen position	Flower inclination	-0.01	0.932
Display size	Flower inclination	0.08	0.601
Nectar width	Nectar depth	0.62	0.001
Pollen position	Nectar depth	-0.03	0.850
Display size	Nectar depth	0.14	0.359
Pollen position	Nectar width	-0.06	0.709
Display size	Nectar width	0.05	0.719
Display size	Pollen position	-0.54	0.001
